# Impact of Cervical Dystonia on Work Productivity: An Analysis From a Patient Registry

**DOI:** 10.1002/mdc3.12238

**Published:** 2015-12-16

**Authors:** Eric S. Molho, Mark Stacy, Patrick Gillard, David Charles, Charles H. Adler, Joseph Jankovic, Marc Schwartz, Mitchell F. Brin

**Affiliations:** ^1^ Albany Medical Center Albany New York USA; ^2^ Duke University Medical Center Durham North Carolina USA; ^3^ Allergan, Inc. Irvine California USA; ^4^ Vanderbilt University Medical Center Nashville Tennessee USA; ^5^ Mayo Clinic Arizona Scottsdale Arizona USA; ^6^ Baylor College of Medicine Houston Texas USA; ^7^ MedNet Solutions, Inc. Minnetonka Minnesota USA; ^8^ University of California Irvine California USA

**Keywords:** cervical dystonia, employment, botulinum toxin

## Abstract

**Background:**

Cervical dystonia is thought to result in high disease burden, but limited information exists on its impact on employment and work productivity. We utilized data from the Cervical Dystonia Patient Registry for the Observation of OnabotulinumtoxinA Efficacy (ClinicalTrials.gov identifier: NCT00836017) to assess the impact of cervical dystonia on employment and work productivity and examine the effect of onabotulinumtoxinA treatments on work productivity.

**Methods:**

Subjects completed a questionnaire on employment status and work productivity at baseline and final visit. Baseline data were examined by severity of cervical dystonia, predominant subtype, presence of pain, prior exposure to botulinum toxin, and/or utility of a sensory trick. Work productivity results at baseline and final visit were compared in subjects who were toxin‐naïve at baseline and received three onabotulinumtoxinA treatments.

**Results:**

Of 1,038 subjects, 42.8% were employed full‐ or part‐time, 6.1% unemployed, 32.7% retired, and 11.8% disabled. Of those currently employed, cervical dystonia affected work status of 26.0%, caused 29.8% to miss work in the past month (mean, 5.1 ± 6.4 days), and 57.8% reported decreased productivity. Half of those unemployed were employed when symptoms began, and 38.5% attributed lost employment to cervical dystonia. Pain, increasing severity, and anterocollis/retrocollis had the largest effects on work status/productivity. Preliminary analyses showed that absenteeism and presenteeism were significantly decreased following onabotulinumtoxinA treatments in the subpopulation that was toxin‐naïve at baseline.

**Conclusions:**

This analysis confirms the substantial negative impact of cervical dystonia on employment, with cervical dystonia‐associated pain being a particularly important driver. OnabotulinumtoxinA treatment appears to improve work productivity.

Cervical dystonia (CD), the most common form of adult‐onset dystonia, is characterized by involuntary contractions of cervical muscles resulting in spasmodic head movements, abnormal neck postures, and/or irregular head tremor.^1^, ^2^, ^3^, ^4^ It affects women more commonly than men, and pain occurs in approximately 75% of patients.^2^, ^5^, ^6^ Remission is rare (in <20% of patients),^2^, ^7^ and most patients ultimately relapse.^2^, ^8^ The onset of disease is most often in the fourth or fifth decade of life,^1^, ^4^ which, for most patients, would be a time of secure employment and peak productivity. Thus, CD patients may be at high risk for economic harm, including loss of employment and reduced productivity. Cross‐sectional, survey‐based studies have addressed the impact of CD specifically on employment,^6^, ^9^, ^10^, ^11^, ^12^ but these involved relatively small sample sizes and/or did not examine factors that contribute to the burden of disease on work productivity. In addition, botulinum toxin (BoNT) is considered to be the first‐line treatment of choice for CD,^1^, ^13^ but there is little information on the ability of treatment to maintain or restore employment status and work productivity.

The Cervical Dystonia Patient Registry for the Observation of OnabotulinumtoxinA Efficacy (CD PROBE) study is the largest of its kind and represents a unique opportunity to characterize the clinical and social aspects of CD.^14^ We report baseline data regarding the impact of CD with the specific aims to (1) determine the frequency and severity of impairment of employment, (2) determine the clinical features associated with impairment of employment, and (3) examine the effect of onabotulinumtoxinA on work productivity.

## Patients and Methods

The full methods of CD PROBE, a prospective, observational registry of CD subjects in the United States, have been previously described.^14^ Subjects were naïve to BoNT, new to the physician's practice, and/or had not received BoNT for ≥16 weeks if previously in a clinical trial. Each center obtained institutional review board approval, and each subject gave written informed consent. Enrolled subjects could receive up to three onabotulinumtoxinA (BOTOX, Allergan, Inc., Irvine, CA) treatment sessions in an open‐label format, with treatment interval, dilution, dosing, injection guidance, and muscles injected with onabotulinumtoxinA at the discretion of the treating physician. The final office visit did not include a treatment.

Baseline demographics and disease characteristics and physician assessments of CD severity and predominant type were collected. Physicians also administered the Toronto Western Spasmodic Torticollis Rating Scale (TWSTRS).^15^ Subject assessments included the Pain Numeric Rating Scale (PNRS)^16^ and a work productivity questionnaire (Fig. S1) that assessed overall employment, lost work days (absenteeism), and lost productivity (presenteeism).^14^

The baseline as‐treated population included those who reported their previous exposure to BoNT treatment, completed treatment session 1, and completed the baseline work productivity questionnaire. Baseline characteristics by employment status were compared using two‐sample *t* tests or one‐way analysis of variance (for continuous measures) and uncorrected chi‐square tests (for categorical measures). In cases where only partial dates were available, the 15th of the month was used when the day was missing, and July 1 was used when the month was missing. The work productivity questionnaire data were examined by baseline factors, including prior toxin exposure, CD severity, CD subtype, presence of pain (defined as a score >0 on the PNRS or TWSTRS Pain subscale), utility of a sensory trick, and time from diagnosis to the first treatment. For the item on the effect of sensory tricks from the TWSTRS Severity subscale, the possible answers of “complete relief by ≥1 tricks” and “partial or only limited relief by tricks” were combined for comparison with “little or no benefit from tricks.”

The impact of CD on employment was measured in three ways in the employed group: work status compared with usual employment level; absenteeism (missed work); and presenteeism (reduced productivity). Employment at baseline (including responses of full‐time, part‐time, self‐employed, or other) was also categorized by age and gender and was compared with employment data from the 2009–2012 U.S. population, as reported by the U.S. Bureau of Labor Statistics,^17^ weighted by the percentage of CD PROBE subjects recruited in each year.

In an exploratory analysis, descriptive statistics were used to compare the results from the work productivity questionnaire at the baseline and final visits in the subpopulation that was toxin‐naïve at baseline. For subjects who reported missed work days at baseline and did not report missed work days at the final visit, 0 days was imputed as the value for the final visit. Similarly, for those who reported reduced work productivity at baseline but not at the final visit, 100% was imputed as the value for the final visit.

## Results

A total of 1,046 subjects were enrolled between January 12, 2009 and August 31, 2012. The baseline as‐treated population included 1,038 subjects who completed the first treatment session, reported whether they had received previous BoNT treatment, and completed the work productivity questionnaire at baseline. Demographics by baseline employment are presented in Table 1. As a whole, this population was an average of 58.0 ± 14.7 years of age, with an age at onset of 49.0 ± 16.7 years, 74.5% female, and 92.4% Caucasian. The majority of subjects (n = 594; 57.2%) were not employed at baseline; however, nearly all subjects (n = 1,000; 96.3%) had been employed at some point in the past. Compared with those who were employed, unemployed subjects were older at baseline (63.2 ± 14.6 vs. 51.1 ± 11.9 years; *P* < 0.0001) and at symptom onset (53.4 ± 17.5 vs. 43.2 ± 13.6 years; *P* < 0.0001). There were also significant differences in education level, marital status, and income. Employed and unemployed subjects otherwise did not differ in terms of gender, race/ethnicity, time from symptom onset to diagnosis, or time from diagnosis to first treatment.

**Table 1:** Baseline demographics and disease characteristics in the overall study population and split by employment status

Characteristic	Overall (N = 1,038)	Employed (n = 444)	Not Employed (n = 594)	*P* Value
Age, years	58.0 ± 14.7	51.1 ± 11.9	63.2 ± 14.6	<0.0001
Gender				0.1363
Female	773 (74.5)	341 (76.8)	432 (72.7)	
Male	265 (25.5)	103 (23.2)	162 (27.3)	
Race/ethnicity				0.5423
Asian	17 (1.6)	8 (1.8)	9 (1.5)	
Black	24 (2.3)	8 (1.8)	16 (2.7)	
Caucasian	959 (92.4)	413 (93.0)	546 (91.9)	
Hispanic	34 (3.3)	15 (3.4)	19 (3.2)	
Other/Native American	4 (0.4)	0 (0)	4 (0.7)	
Employment status				<0.0001
Employed full‐time	309 (29.8)	309 (69.6)	0 (0)	
Employed part‐time	67 (6.5)	67 (15.1)	0 (0)	
Self‐employed	61 (5.9)	61 (13.7)	0 (0)	
Disabled	123 (11.8)	0 (0)	123 (20.7)	
Retired	339 (32.7)	0 (0)	339 (57.1)	
Other	139 (13.4)	7 (1.6)	132 (22.2)^a^	
Education level				<0.0001
Less than a high school diploma	41 (3.9)	6 (1.4)	35 (5.9)	
High school graduate/some college	518 (49.9)	174 (39.2)	344 (57.9)	
Associate/bachelor's degree	315 (30.3)	178 (40.1)	137 (23.1)	
Advanced degree (masters, doctoral, professional)	147 (14.2)	82 (18.5)	65 (10.9)	
Other	17 (1.6)	4 (0.9)	13 (2.2)	
Marital status				<0.0001
Single	126 (12.1)	69 (15.5)	57 (9.6)	
Married	638 (61.5)	286 (64.4)	352 (59.3)	
Divorced	145 (14.0)	56 (12.6)	89 (15.0)	
Living together	3 (0.3)	3 (0.7)	0 (0)	
Separated	14 (1.3)	6 (1.4)	8 (1.3)	
Widowed	108 (10.4)	21 (4.7)	87 (14.6)	
Other	4 (0.4)	3 (0.7)	1 (0.2)	
Annual income, USD				<0.0001
<$25,000	208 (20.0)	43 (9.7)	165 (27.8)	
$25,001–50,000	157 (15.1)	77 (17.3)	80 (13.5)	
$50,001–75,000	107 (10.3)	52 (11.7)	55 (9.3)	
$75,001–100,000	51 (4.9)	31 (7.0)	20 (3.4)	
$100,001–125,000	33 (3.2)	19 (4.3)	14 (2.4)	
$125,001–150,000	27 (2.6)	20 (4.5)	7 (1.2)	
>$150,000	30 (2.9)	24 (5.4)	6 (1.0)	
Did not disclose	425 (40.9)	178 (40.1)	247 (41.6)	
Age at symptom onset, years	49.0 ± 16.7	43.2 ± 13.6	53.4 ± 17.5	<0.0001
Time from CD onset to diagnosis	5.0 ± 8.1	5.2 ± 7.2	4.9 ± 8.8	0.5011
Time from CD diagnosis to treatment	1.1 ± 4.5	0.9 ± 3.8	1.3 ± 5.0	0.1248

Data are presented as mean ± SD or n (%). The number of employed subjects was obtained by adding those who indicated their employment status as full‐time, part‐time, self‐employed, or other on the baseline demographics questionnaire.

a Includes homemaker, never employed, student, and unemployed.

SD, standard deviation; USD, United States dollars.

**Figure 1:**
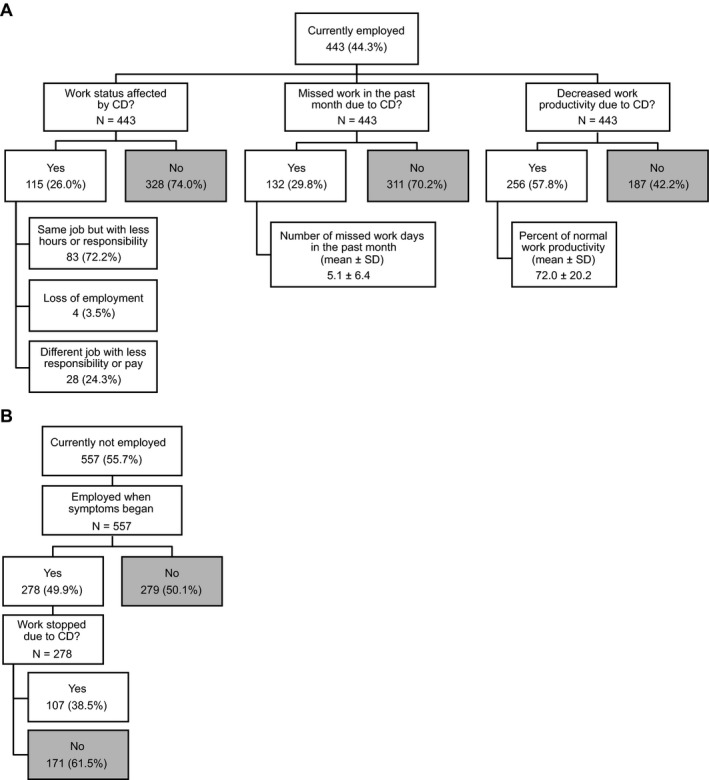
(A) Baseline work status, absenteeism, and productivity in CD PROBE subjects who were currently employed at baseline and (B) baseline effect of CD on employment in subjects not currently employed at baseline, both in the as‐treated baseline population that had ever been employed (N = 1,000).

Figure 1 details the effects of CD on employment at baseline in the as‐treated population that had ever been employed (N = 1,000). Of those subjects still able to work despite CD, 26.0% experienced some change in employment level, 29.8% missed work in the month leading up to study participation (absenteeism), and 57.8% reported reduced productivity due to CD (presenteeism). The mean number of days missed in those subjects reporting absenteeism was 5.1 ± 6.4 days/month. In the subjects with reduced productivity, the mean percent of normal productivity was 72.0%, which equates to a loss of 11 hours of a 40‐hour work week. Half of those unemployed at baseline had been employed at the onset of CD symptoms, and 38.5% of those indicated that CD was the cause of job loss.

**Figure 2:**
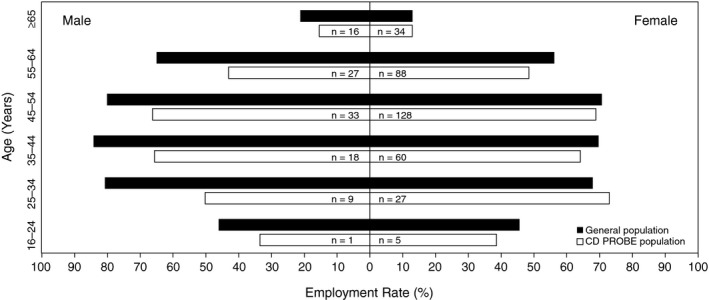
Employment by age and gender in the CD PROBE and 2009–2012 U.S. populations. For the CD PROBE population, responses of full‐time, part‐time, self‐employed, and other were considered employed, with the number employed indicated within the bars. General population values were from the U.S. Bureau of Labor and Statistics employment population ratio (weighted based on years of recruitment for CD PROBE).

Figure 2 compares subjects employed at baseline, categorized by age and gender, with employment data from the 2009–2012 U.S. population.^17^ For all age groups, men in the study cohort were employed at a lower rate than age‐matched figures for the general population. While employment in the female study population was lower than in the general population in some age groups, the differences were smaller than those seen for the men, and no obvious trend was apparent. Women in the study were employed at equal or higher rates to the general population in the ≥65 and in the 25‐ to 34‐year age groups.

Table 2 details associations between the clinical features of CD and the relevant employment impact assessed at baseline. The strongest associations were for pain (>0 on the PNRS or TWSTRS), with significant associations for lost employment due to CD, work status changes, absenteeism, and reduced productivity. The predominant type of CD was also associated with employment outcomes, with anterocollis more likely to be associated with work stoppage due to CD and absenteeism and retrocollis the least likely to be currently employed. Increasing severity of CD, as measured by the physician, was associated with fewer currently employed subjects and more who had stopped work due to CD or had reduced productivity. The presence of a useful sensory trick was associated with current employment, but none of the other outcomes (Table S1). The degree to which first treatment was delayed after diagnosis, with a cutoff of <1 or ≥1 year, did not bear any statistical associations with the effects of CD on employment, with the exception that those with <1 year from diagnosis to first treatment were less likely to be employed when symptoms began (Table S1).

**Table 2:** Effect of CD on work productivity and employment status examined by the clinical features of pain and predominant CD subtype

	**Pain (PNRS)** a			**Pain (TWSTRS)** a			**Subtype** a				
	No Pain (n = 115)	Pain (n = 922)	*P* Value	No Pain (n = 86)	Pain (n = 949)	*P* Value	Anterocollis (n = 59)	Laterocollis (n = 403)	Retrocollis (n = 55)	Torticollis (n = 493)	*P* Value
**Currently employed**	48.7% (55/113)	43.7% (387/886)	0.3142	46.5% (40/86)	44.2% (403/911)	0.6849	37.9% (22/58)	45.7% (176/385)	16.0% (8/50)	47.1% (226/480)	0.0003
**Employed when symptoms began**	43.1% (25/58)	50.7% (253/499)	0.2734	45.7% (21/46)	50.0% (254/508)	0.5722	44.4% (16/36)	45.0% (94/209)	52.4% (22/42)	52.8% (134/254)	0.3515
**Work stopped due to CD**	12.0% (3/25)	41.1% (104/253)	0.0043	14.3% (3/21)	40.2% (102/254)	0.0190	68.8% (11/16)	31.9% (30/94)	45.5% (10/22)	40.3% (54/134)	0.0392
**Employment status affected by CD**			0.0364			0.0489					0.0008
No	89.1% (49/55)	71.8% (278/387)		92.5% (37/40)	72.2% (291/403)		68.2% (15/22)	68.8% (121/176)	50.0% (4/8)	79.6% (180/226)	
Yes: different job with less responsibility or pay	5.5% (3/55)	6.5% (25/387)		2.5% (1/40)	6.7% (27/403)		9.1% (2/22)	5.7% (10/176)	12.5% (1/8)	6.2% (14/226)	
Yes: loss of employment	0.0% (0/55)	1.0% (4/387)		0.0% (0/40)	1.0% (4/403)		9.1% (2/22)	0.6% (1/176)	0.0% (0/8)	0.4% (1/226)	
Yes: same job, reduced hours or responsibility	5.5% (3/55)	20.7% (80/387)		5.0% (2/40)	20.1% (81/403)		13.6% (3/22)	25.0% (44/176)	37.5% (3/8)	13.7% (31/226)	
**Decreased work productivity due to CD (presenteeism)**	32.7% (18/55)	61.5% (238/387)	<0.0001	30.0% (12/40)	60.5% (244/403)	0.0002	72.7% (16/22)	58.0% (102/176)	75.0% (6/8)	56.6% (128/226)	0.3813
Percentage of normal work productivity	84.3 ± 18.0	71.0 ± 20.0	0.0070	84.2 ± 21.0	71.4 ± 20.0	0.0607	70.3 ± 23.6	71.9 ± 19.2	75.0 ± 14.8	72.4 ± 20.6	0.9506
**Missed work in the past month due to CD (absenteeism)**	3.6% (2/55)	33.6% (130/387)	<0.0001	2.5% (1/40)	32.5% (131/403)	<0.0001	45.5% (10/22)	34.1% (60/176)	37.5% (3/8)	23.9% (54/226)	0.0430
**Number of missed work days in past month**	3.5 ± 3.5	5.1 ± 6.4	0.6275	6.0 ± NA	5.1 ± 6.4	NA	9.6 ± 9.5	4.1 ± 5.0	3.7 ± 2.3	5.5 ± 7.1	0.2975

Data are presented as percentage of those answering a particular question in the affirmative out of all those answering the question, except where otherwise noted. *P* values indicate significant differences in distribution within each group; shading indicates nonsignificance.

a Of the 1,038 subjects in the analysis population, data were missing for 1 subject on the PNRS, for 3 subjects on the TWSTRS, and for 28 subjects on subtype.

NA, not applicable.

**Figure 3:**
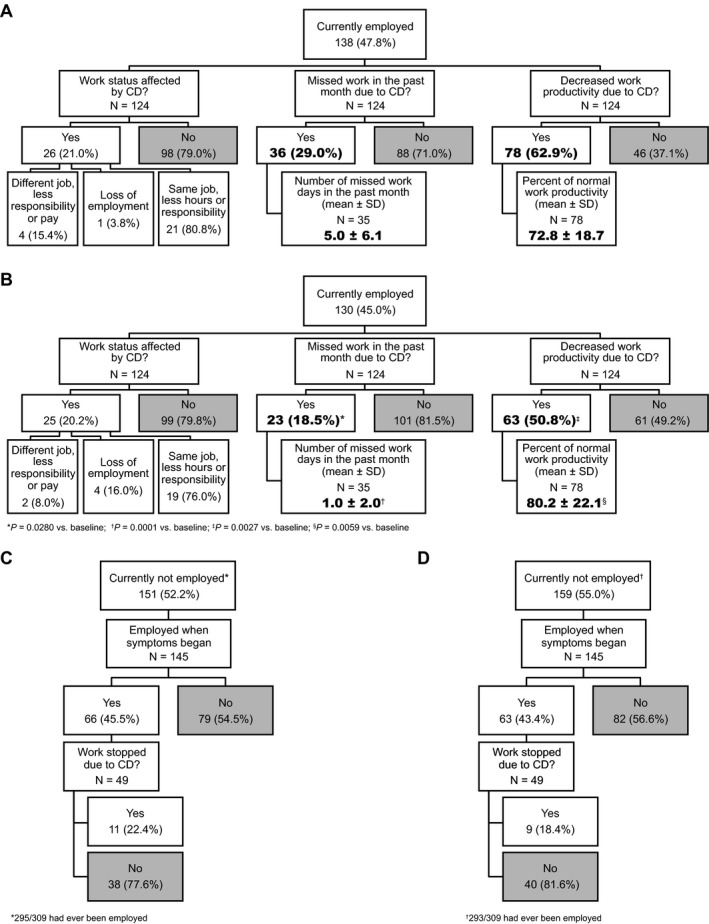
For the population that was toxin‐naïve at baseline, (A) baseline work status, absenteeism, and productivity in subjects who were employed at baseline, (B) final work status, absenteeism, and productivity in subjects who were employed at baseline, (C) baseline effect of CD on employment in subjects not currently employed at baseline, and (D) final effect of CD on employment in subjects not currently employed at baseline. SD, standard deviation.

The baseline work productivity questionnaire data in the toxin‐naïve subpopulation (Fig. 3) were overall very similar to that of the baseline as‐treated population (Fig. 1). Employment did not differ between the toxin‐naïve and previously treated groups, except that the previously treated group was more likely to have been employed at the onset of CD symptoms and to have stopped work due to CD (Table S1). For those who were toxin‐naïve and currently employed at baseline, the percentage of subjects who had missed work in the past month due to CD significantly decreased from 29.0% at baseline to 18.5% at final visit (*P* = 0.0280), and the number of missed work days also significantly decreased from 5.0 ± 6.1 to 1.0 ± 2.0 (*P* = 0.0001; Fig. 3A,B). Similarly, the percentage of subjects reporting decreased work productivity due to CD significantly decreased from 62.9% at baseline to 50.8% at final visit (*P* = 0.0027), with a significant increase in the mean percent of normal work productivity from 72.8% to 80.2% (*P* = 0.0059).

## Discussion

This analysis of the data from the CD PROBE study, the largest prospective study of subjects with CD, represents a unique opportunity to explore important clinical and social aspects of CD in more detail than previously possible. The most important findings presented in this analysis are a confirmation of the impact of CD on several aspects of employment and the close association of pain with these adverse outcomes. In addition, there is suggestive evidence that particular subtypes of CD, such as predominant anterocollis or retrocollis, may be more problematic in terms of employment. In an exploratory analysis of the subpopulation that was toxin‐naïve at baseline, absenteeism and presenteeism were found to significantly improve after treatments with onabotulinumtoxinA. The results confirm that CD is frequently associated with adverse consequences on employment, including absenteeism, reduced productivity, and loss of employment, which are consistent with previous smaller studies.^6^, ^11^, ^18^, ^19^, ^20^, ^21^

It was previously suggested that job impairment due to CD is age specific, which may be more accurately viewed as a problem of early retirement due to CD disability, with a distinct dropoff of employment in the Finnish CD population starting at around age 45.^20^ In contrast, the CD PROBE data show a gender disparity in employment levels, with fewer employed males in this CD cohort compared with males in the general U.S. population across all ages. Employment rates for females in this CD cohort were closer to those females in the general U.S. population for all ages and met or exceeded these levels in the 25‐ to 34‐ and ≥65‐year age groups. Although the meaning of this gender disparity is not known, one could speculate that selection bias played a role. Employed men with CD may be less likely to volunteer for research studies or switch to a new provider for injections compared to employed women with CD. Another possibility that was not addressed by this study is that type of employment (manual labor vs. sedentary or office work) may have been unequally distributed in this CD population, with men more represented in jobs that would be less tolerant of CD‐related impairments.

Data from this large population also duplicate the previously observed association between CD pain and negative impact on employment and productivity. A prior study found that neck pain was significantly associated with altered employment, reduced productivity, and the likelihood of applying for disability benefits, but type of employment, spasmodic versus fixed dystonia, and duration of symptoms before first treatment with BoNT were not associated with these outcomes.^6^ In the current study, expanding beyond previous work, consistent associations were found between the presence of pain, as measured by the PNRS and TWSTRS scales, for nearly every measure of employment impact analyzed. Less consistent, but potentially meaningful, associations were also observed between the predominant type of CD and employment outcomes, where anterocollis and retrocollis seemed to be more frequently associated with adverse impacts on employment than predominant torticollis. Finally, in contrast with a previous finding,^10^ these data do not show an association between the lack of a useful sensory trick and adverse employment outcomes. However, it should be noted that the present analysis simply tested for a statistical difference between the proportions of subjects with each clinical feature with regard to each employment outcome. Magnitude, directionality, and relative impact compared with other clinical features cannot be gleaned from this analysis.

In order to explore the effect of onabotulinumtoxinA treatments on work productivity, the subpopulation that was toxin‐naïve at baseline was examined. For those who were currently employed, significant decreases from baseline were observed after onabotulinumtoxinA treatments in the percentage that missed work in the past month due to CD (absenteeism), number of missed work days, and percentage of subjects with decreased work productivity due to CD (presenteeism); the percentage of normal work productivity significantly increased from baseline. Though these data are exploratory, they do point to onabotulinumtoxinA as an available treatment that may improve work productivity in patients with CD. Work status affected by CD did not significantly change from baseline to final assessment, but it is expected that work status would not be affected for this chronic condition over the relatively short time span of three treatments. Similarly, for those who were currently unemployed, there was little change from baseline to final visit in their employment or whether they had stopped work due to CD. Again, changes would not be expected given the short‐term treatment span and historical nature of the questions. Indeed, a Norwegian study in which subjects had been treated with BoNT for a median of 5 years showed an increase in the employment rate from 47% to 65% in those ≤55 years of age.^18^

There are a few caveats to keep in mind regarding the present study. The demographics of this cohort of CD subjects are typical of those observed in previous cross‐sectional analyses,^2^, ^4^, ^9^ but is limited to subjects with CD who are seeking treatment with BoNT injections and have been referred to a subspecialty clinic for care. At baseline, the previously treated group may not be representative of the BoNT‐treated CD population in general, since our cohort only included those who previously were treated by physicians other than the treating investigator in the study, and in many cases, this may mean prior treatment by a less‐specialized or less‐experienced practitioner. This is also a group of subjects that may have been more likely to be dissatisfied with previous treatments, since they either sought out or were willing to switch injectors to be included in the study. Likewise, the toxin‐naïve group may not be representative of the untreated CD patient population in general, as this group is not inclusive of patients who had never sought medical care for their condition, have never been diagnosed, do not have health insurance, and/or those that are simply too mildly affected to agree to a treatment involving multiple intramuscular injections. Nevertheless, the CD PROBE study provided the opportunity to characterize the baseline burden of CD on employment status and work productivity in this large study population and prospectively follow these important outcomes over three full treatment cycles with onabotulinumtoxinA.

## Author Roles

(1) Research Project: A. Conception, B. Organization, C. Execution; (2) Statistical Analysis: A. Design, B. Execution, C. Review and Critique; (3) Manuscript: A. Writing the First Draft, B. Review and Critique.

E.S.M.: 1B, 1C, 3A

M.St.: 1A, 1B, 1C, 3B

P.G.: 1B, 1C, 3B

D.C.: 1A, 1B, 1C, 3B

C.H.A.: 1A, 1B, 1C, 3B

J.J.: 1A, 1B, 1C, 3B

M.Sc.: 2A, 2B, 2C, 3B

M.F.B.: 1A, 1B, 1C, 3B

## Disclosures

**Funding Sources and Conflicts of Interest:** This study and its analysis were sponsored by Allergan, Inc. (Irvine, CA). E.S.M., M.St., C.H.A., and J.J. are study investigators for Allergan, Inc., and M.St., C.H.A., and J.J. have received compensation for consulting services for Allergan, Inc. M.Sc. is an employee of MedNet Solutions, Inc., which provides statistical analysis services to Allergan, Inc. P.G. and M.F.B. are employees of Allergan, Inc. Vanderbilt University receives income from grants and/or contracts with Allergan, Inc. for research and educational programs led by D.C. D.C. receives income from Allergan, Inc. for consulting services.

**Financial Disclosures for previous 12 months:** E.S.M. received compensation from Lundbeck, US World Meds, and Merz for consulting services; he has received research support from Merz, Cure HD Network, Teva, Allergan, Prana Biotechnologies, the National Institutes of Health (NIH), the Michael J. Fox Foundation for Parkinson's Research (MJFF), Auspex, and Kyowa Hakko Kirin, and he received compensation from US World Meds for education services. M.St. has received grant/research support from the MJFF, NIH, and Parkinson Study Group; served as a consultant for Acorda, Allergan, Chelsea, General Electric, Genzyme, Eli Lilly, Merz, Osmotica, Pfizer, ProStraken, SK Life Sciences, UCB, and Vanda; has served on a protocol steering committee for Allergan; has received royalties from Informa Press; and is an editorial board member for *Journal of Clinical Investigation*. Vanderbilt University receives income from grants and contracts with Allergan, Ipsen, Merz, and Medtronic for research or educational programs led by D.C. D.C. receives income for Allergan, Concert, Ipsen, Merz, and Medtronic for education or consulting services. C.H.A. received compensation from Allergan, Eli Lilly, Merz, Lundbeck, Teva, and Acadia for consulting services. J.J. received research and Center of Excellence Grants from Adamas Pharmaceuticals, Inc, Allergan, Inc, Auspex Pharmaceuticals, Inc, Civitas Therapeutics, Huntington's Disease Society of America, Huntington Study Group, Ipsen Limited, Kyowa Haako Kirin Pharma, Inc, Lundbeck Inc, Medtronic, Merz Pharmaceuticals, MJFF, NIH, National Parkinson Foundation, Omeros Corporation, Parkinson Study Group, Pfizer, Prothena Biosciences Inc, Psyadon Pharmaceuticals, Inc, St. Jude Medical, and Teva Pharmaceutical Industries Ltd; has served as a consultant or an advisory committee member for Adamas Pharmaecuticals, Inc, Allergan, Inc, Auspex Pharmaceuticals, Inc, Lundbeck Inc, and Teva Pharmaceutical Industries Ltd; and has received royalties from Cambridge, Elsevier, Future Science Group, Hodder Arnold, Lippincott Williams and Wilkins, and Wiley‐Blackwell. M.Sc. is an employee of MedNet Solutions. P.G. and M.F.B. are employees of Allergan, Inc. and receive salary, stock, and stock options from Allergan, Inc.

## Supporting information


**Figure S1.** CD PROBE work productivity questionnaire.

**Table S1.** Effect of CD on work productivity and employment status examined by the clinical features of CD severity, prior toxin exposure at baseline, time from CD diagnosis to first treatment, and utility of a sensory trick
