# Supplementary material for: Impact of Cervical Dystonia on Work Productivity: An Analysis From a Patient Registry
Source: Mov Disord Clin Pract. 2015 Dec 16;3(2):130–8. doi: 10.1002/mdc3.12238 (PMC5064605; doi:10.1002/mdc3.12238)
Supplement: Supplementary file 1 — Figure S1. CD PROBE work productivity questionnaire. [file MDC3-3-130-s001.pdf]

Healthcare Utilization & Work Productivity

Patient Registration ID: ☐☐☐ Site ID ☐☐ Patient ID ☐☐☐ **Patient Initials** (FML)

Work Status and Work Productivity in Cervical Dystonia:

Have you ever been employed (including self-employed)? ☐ Yes ☐ No

STOP: If you have never been employed, please stop here

Are you currently employed (including being self-employed)? ☐ Yes ☐ No

If you are **NOT** currently employed answer the following:

1. Were you employed (including being self-employed) when symptoms of cervical dystonia began?
   ☐ Yes ☐ No
2. If you were employed when you were diagnosed with CD, did the CD cause you to stop working?
   ☐ Yes ☐ No

If you **ARE** currently employed answer the following:

1. Has your employment status been affected by cervical dystonia?
   - ☐ No
   - ☐ Same job, reduced hours or responsibility
   - ☐ Different job with less responsibility or pay
   - ☐ Loss of employment
2. Over the past month, have you missed work due to your cervical dystonia?
   ☐ Yes ☐ No
   If yes, number of days: _____
3. Does cervical dystonia decrease your productivity at work? That is, does your disease impact how effective you are or can be at work or how much work you can complete?
   ☐ Yes ☐ No
   If yes, relatively speaking, how productive are you at work? ____ %
   (100% = normal, 0% = no work gets done)
