# Supplementary material for: Impact of Cervical Dystonia on Work Productivity: An Analysis From a Patient Registry
Source: Mov Disord Clin Pract. 2015 Dec 16;3(2):130–8. doi: 10.1002/mdc3.12238 (PMC5064605; doi:10.1002/mdc3.12238)
Supplement: Supplementary file 2 — Table S1. Effect of CD on work productivity and employment status examined by the clinical features of CD severity, prior toxin exposure at baseline, time from CD diagnosis to first treatment, and utility of a sensory trick [file MDC3-3-130-s002.docx]

**Supplemental table 1** Effect of CD on work productivity and employment status examined by the clinical features of CD severity, prior toxin exposure at baseline, time from CD diagnosis to first treatment, and utility of a sensory trick

|  | Severity* | | | | Prior toxin exposure | | | Time from diagnosis to first treatment | | | Utility of sensory trick* | | |  |
| --- | --- | --- | --- | --- | --- | --- | --- | --- | --- | --- | --- | --- | --- | --- |
|  | Mild  (n = 344) | Moderate  (n = 547) | Severe  (n = 146) | *P* value | Naïve  (n = 660) | Non-naïve  (n = 378) | *P* value | <1 year  (n = 899) | ≥1 year  (n = 139) | *P* value | Complete/ partial relief  (n = 744) | Little/no relief  (n = 291) | *P* value | |
| Currently employed | 50.9%  (171/336) | 42.2%  (222/526) | 36.5%  (50/137) | 0.0060 | 45.3%  (289/638) | 42.5%  (154/362) | 0.3991 | 44.4%  (384/865) | 43.7%  (59/135) | 0.8808 | 47.8%  (343/717) | 35.7%  (100/280) | 0.0005 | |
| Employed when symptoms began | 41.8%  (69/165) | 50.3%  (153/304) | 63.2%  (55/87) | 0.0052 | 41.8%  (146/349) | 63.5%  (132/208) | <0.0001 | 47.8%  (230/481) | 63.2%  (48/76) | 0.0129 | 50.8%  (190/374) | 47.2%  (85/180) | 0.4299 | |
| Work stopped due to CD | 31.9%  (22/69) | 35.3%  (54/153) | 56.4%  (31/55) | 0.0094 | 30.1%  (44/146) | 47.7%  (63/132) | 0.0026 | 39.1%  (90/230) | 35.4%  (17/48) | 0.6305 | 36.3%  (69/190) | 42.4%  (36/85) | 0.3410 | |
| Employment status affected by CD |  |  |  | 0.8130 |  |  | 0.3516 |  |  | 0.1794 |  |  | 0.6656 | |
| No | 77.8%  (133/171) | 71.6%  (159/222) | 72.0%  (36/50) |  | 74.7%  (216/289) | 72.7%  (112/154) |  | 75.3%  (289/384) | 66.1%  (39/59) |  | 75.2%  (258/343) | 70.0%  (70/100) |  | |
| Yes: different job with less responsibility or pay | 5.3%  (9/171) | 6.8%  (15/222) | 8.0%  (4/50) |  | 4.8%  (14/289) | 9.1%  (14/154) |  | 5.5%  (21/384) | 11.9%  (7/59) |  | 6.4%  (22/343) | 6.0%  (6/100) |  | |
| Yes: Loss of employment | 0.6%  (1/171) | 0.9%  (2/222) | 2.0%  (1/50) |  | 1.0%  (3/289) | 0.6%  (1/154) |  | 1.0%  (4/384) | 0.0%  (0/59) |  | 0.9%  (3/343) | 1.0%  (1/100) |  | |
| Yes: Same job, reduced hours or responsibility | 16.4%  (28/171) | 20.7%  (46/222) | 18.0%  (9/50) |  | 19.4%  (56/289) | 17.5%  (27/154) |  | 18.2%  (70/384) | 22.0%  (13/59) |  | 17.5%  (60/343) | 23.0%  (23/100) |  | |
| Decreased work productivity due to CD (presenteeism) | 49.7%  (85/171) | 63.5%  (141/222) | 60.0%  (30/50) | 0.0217 | 58.8%  (170/289) | 55.8%  (86/154) | 0.5454 | 57.0%  (219/384) | 62.7%  (37/59) | 0.4108 | 57.4%  (197/343) | 59.0%  (59/100) | 0.7803 | |
| Percentage of normal work productivity | 75.2 ± 20.3 | 70.4 ± 19.8 | 69.9 ± 21.1 | 0.1965 | 70.9 ± 20.4 | 74.0 ± 19.6 | 0.2524 | 71.9 ± 20.3 | 72.2 ± 19.9 | 0.9394 | 72.9 ± 20.2 | 68.7 ± 19.8 | 0.1495 | |
| Missed work in the past month due to CD (absenteeism) | 26.9%  (46/171) | 32.9%  (73/222) | 26.0%  (13/50) | 0.3604 | 31.1%  (90/289) | 27.3%  (42/154) | 0.3965 | 30.2%  (116/384) | 27.1%  (16/59) | 0.6290 | 29.4%  (101/343) | 31.0%  (31/100) | 0.7650 | |
| Number of missed work days in past month | 3.8 ± 6.0 | 5.6 ± 6.4 | 7.3 ± 7.0 | 0.1860 | 5.5 ± 6.9 | 4.4 ± 5.2 | 0.3465 | 5.1 ± 6.6 | 5.5 ± 4.7 | 0.7506 | 4.7 ± 5.8 | 6.5 ± 7.9 | 0.2621 | |

Data are presented as percentage of those answering a particular question in the affirmative out of all those answering the question, except where otherwise noted.

*P* values indicate significant differences in distribution within each group; shading indicates nonsignificance.

*Out of the 1,038 subjects in the analysis population, data were missing for 1 subject on severity and for 3 subjects on utility of a sensory trick.

CD, cervical dystonia.
